# Avaliação Ecocardiográfica com *Strain* do Envolvimento Miocárdico em Pacientes com Dor Torácica Contínua após Infecção por COVID-19

**DOI:** 10.36660/abc.20220287

**Published:** 2022-12-20

**Authors:** Emre Özdemir, Uğur Karagöz, Sadık Volkan Emren, Sedat Altay, Nihan Kahya Eren, Selin Özdemir, Mehmet Tokaç

**Affiliations:** 1 Faculdade de Medicina Atatürk Research and Training Hospital Katip Çelebi University Izmir Turquia Departamento da Cardiologia, Faculdade de Medicina, Atatürk Research and Training Hospital, Katip Çelebi University, Izmir – Turquia; 2 Departamento da Cardiologia Torbalı Goverment Hospital Izmir Turquia Departamento da Cardiologia, Torbalı Goverment Hospital, Izmir – Turquia; 3 Departamento da Radiologia Atatürk Research and Training Hospital Izmir Turquia Departamento da Radiologia, Atatürk Research and Training Hospital, Izmir – Turquia; 4 Departamento de Doenças Infecciosas Bozyaka Research and Training Hospital Izmir Turquia Departamento de Doenças Infecciosas, Bozyaka Research and Training Hospital, Izmir – Turquia

**Keywords:** COVID-19, Ecocardiografia, Miocardite

## Abstract

**Fundamento:**

Tem surgido uma nova manifestação clínica chamada pós-COVID ou COVID longa (COVID p/l) após a fase aguda da COVID-19. COVID p/l pode levar à lesão miocárdica com problemas cardíacos subsequentes. Diagnosticar esses pacientes de forma rápida e simples é cada vez mais importante devido ao número crescente de pacientes com COVID p/l.

**Objetivos:**

Comparamos os parâmetros de ecocardiografia com *strain* (ES) de pacientes que apresentaram dor torácica atípica e achados de sequelas de miocardite na ressonância magnética cardíaca (RMC). Nosso objetivo foi investigar o valor da ES para detecção de envolvimento miocárdico em pacientes com COVID p/l.

**Métodos:**

Foram incluídos um total de 42 pacientes. Nossa população foi separada em 2 grupos. O grupo RMC(-) (n = 21) não apresentou sequelas miocárdicas na RMC, enquanto o grupo RMC(+) apresentou sequelas miocárdicas na RMC (n = 21). O valor preditivo da ES para miocardite também foi avaliado por análise multivariada ajustada por idade. Valores de p < 0,05 foram considerados estatisticamente significativos.

**Resultados:**

Quando comparado com a fração de ejeção do ventrículo esquerdo (FEVE), o *strain* longitudinal global (SLG) e o *strain* circunferencial global (SCG) tiveram uma relação mais forte (FEVE, p = 0,05; SLG, p < 0,001; SCG, p < 0,001) com envolvimento miocárdico associado à COVID p/l. SLG < 20,35 apresentou sensibilidade de 85,7% e especificidade de 81%; SCG < 21,35 apresentou sensibilidade de 81% e especificidade de 81% como valores diagnósticos para sequelas miocárdicas detectadas com RMC. Enquanto não houve diferença entre os grupos quanto aos marcadores inflamatórios (proteína C-reativa, p = 0,31), houve diferença entre os marcadores bioquímicos, que são indicadores de envolvimento cardíaco (peptídeo natriurético cerebral, p < 0,001).

**Conclusão:**

A ES é mais útil do que a ecocardiografia tradicional para diagnosticar com rapidez e precisão, a fim de não atrasar o tratamento na presença de envolvimento miocárdico.

## Introdução

Em março de 2020, a Organização Mundial da Saúde declarou o surto do novo coronavírus uma pandemia global. Agora sabemos que a COVID-19 causa não apenas pneumonia viral, mas também complicações cardíacas, vasculares, cerebrais, hepáticas e renais, constituindo uma doença multissistêmica complexa.^1 , 2^ Na fase aguda, o envolvimento cardiovascular é causado por lesão viral direta do miocárdio, múltiplas lesões inflamatórias por tempestade de citocinas, disfunção endotelial por vasculite, desestabilização de placas coronarianas existentes, tromboembolismo pulmonar, microtrombogênese e lesão por hipoxemia.^3 , 4^

No entanto, algumas pessoas ainda apresentam sintomas, mesmo após se recuperarem da COVID-19, e isto é chamado de síndrome pós-COVID ou COVID longa (COVID p/l).^5^ Em algumas séries, a dor torácica foi relatada em quase 20% dos pacientes após a recuperação da COVID-19.^6^ O mecanismo da dor torácica ainda não está claro, mas pode estar relacionado aos efeitos de longo prazo da COVID-19 no miocárdio.^7^ A ressonância magnética cardíaca (RMC) pode desempenhar um papel na avaliação dessa síndrome.^8^

Embora a ecocardiografia com *strain* (ES) não seja um dos procedimentos de rotina utilizados pelos cardiologistas, alguns estudos têm demonstrado que parâmetros de ES baixos podem detectar a progressão da doença miocárdica antes que os parâmetros ecocardiográficos tradicionais se agravem.^9 , 10^ Parâmetros de ES baixos podem ser detectados durante a fase aguda da COVID-19 independentemente do estado clínico e de ecocardiografia tradicional e podem se resolver durante o período de acompanhamento.^11 - 13^ No entanto, não existem dados suficientes sobre a importância dos parâmetros de ES no exame de pacientes com COVID p/l.

No presente estudo, avaliamos os parâmetros ES de pacientes que sofreram de dor torácica atípica após se recuperarem completamente da COVID-19. Em seguida, comparamos esses parâmetros com os achados da RMC de sequelas de miocardite e investigamos o valor da ES para detectar envolvimento miocárdico em pacientes com COVID p/l.

## Materiais e métodos

### Seleção de Pacientes

No presente estudo, selecionamos retrospectivamente um total de 222 pacientes submetidos à avaliação de RMC por qualquer indicação entre fevereiro de 2020 e dezembro de 2021 em um único centro. Desses pacientes, os seguintes dados foram selecionados a partir de registros hospitalares: o período entre a fase aguda da COVID-19 e a avaliação da RMC, história cardíaca prévia e presença de exames cardíacos (tomografia computadorizada coronariana, cintilografia de perfusão miocárdica, teste ergométrico) para excluir dor torácica associada à doença arterial coronariana e queixas de dor torácica contínua.

**Figura 1:**
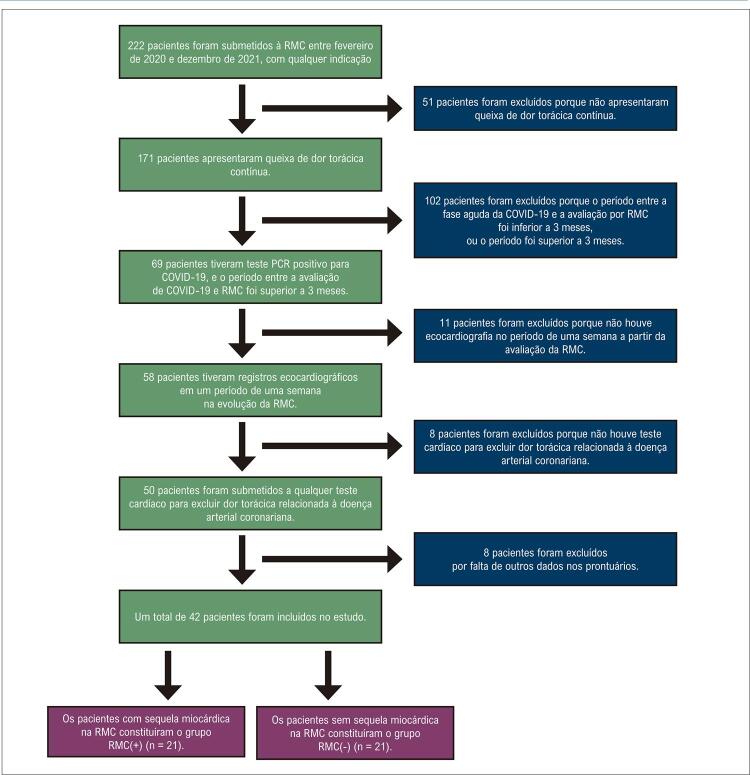
Fluxograma do estudo. RMC: ressonância magnética cardíaca.

Foram excluídos 180 pacientes porque: 1) o período entre a fase aguda da COVID-19 e a avaliação por RMC foi inferior a 3 meses ou o período foi superior a 3 meses, mas não houve teste PCR positivo para COVID-19 (n = 102); 2) sem dor torácica contínua (n = 51); 3) não foi possível realizar a ecocardiografia no período de uma semana a partir da avaliação da RMC (n = 11); 4) ausência de exame cardíaco para excluir dor torácica relacionada à doença arterial coronariana (n = 8); 5) falta de outros dados nos registros hospitalares (n = 8), conforme apresentado na Figura 1 .

Os pacientes foram questionados sobre seus sintomas do período de infecção aguda por COVID-19 durante a admissão com dor torácica contínua. Todos os pacientes apresentaram febre, tosse e dispneia leve, sem necessidade de internação, e nenhum deles descreveu dor torácica durante a fase aguda da COVID-19.

Um total de 42 pacientes que se queixaram de dor torácica que continuou após a recuperação da COVID-19 e tiveram RMC nos registros hospitalares foram inscritos. Nenhum paciente apresentava outras comorbidades. Foram registrados o hemograma de rotina, exames bioquímicos, parâmetros de *strain* (deformação) e parâmetros ecocardiográficos tradicionais de todos os pacientes. Esses pacientes foram divididos em dois grupos de acordo com os achados da RMC compatíveis com sequelas de miocardite. As sequelas miocárdicas foram detectadas como um padrão de realce tardio com gadolínio (RTG) subepicárdico ou da parede média que estava predominantemente localizada nos segmentos basal a médio-lateral do ventrículo esquerdo.

### Coleta de dados

Foram coletados os dados de registros hospitalares, incluindo hemoglobina sérica (hemoglobina), plaquetas, glóbulos brancos, neutrófilos, contagem de linfócitos, creatinina, taxa de filtração glomerular (TFG), proteína C-reativa, peptídeo natriurético cerebral (BNP), níveis de troponina I cardíaca, pressão arterial sistólica (PAS), pressão arterial diastólica (PAD) e índice de massa corporal (IMC).

Todos os dados ecocardiográficos foram obtidos usando uma máquina de ecocardiografia padrão EPIQ 7C (Philips Medical Imaging, Eindhoven, Holanda). Foram avaliados o diâmetro diastólico do ventrículo esquerdo (DDVE), diâmetro sistólico do ventrículo esquerdo (DSVE), diâmetro do átrio esquerdo, diâmetro do septo interventricular e diâmetro da parede posterior, ondas de influxo mitral como a onda precoce de pico (E) e a onda de enchimento tardio (A), as ondas de Doppler tecidual do anel mitral como as velocidades sistólica (s’), diastólica precoce (e’) e diastólica tardia (a’) do anel mitral. A fração de ejeção do ventrículo esquerdo (FEVE) foi medida pelo método biplano de Simpson.

### Avaliação ecocardiográfica com strain

Foram aceitos dados ecocardiográficos adequados, com registros salvos ao final da expiração, adquiridos a partir do pico da onda R, e todas as janelas apicais de 4, 3 e 2 câmaras, bem como o eixo curto paraesternal dos níveis basal, médio-ventricular e apical, avaliados a uma frequência de 50 a 90 quadros por segundo. Foram analisadas as médias de 3 ciclos cardíacos. Os parâmetros de deformação de todos os segmentos foram calculados pelo software (QLAB, Philips). Subsequentemente, foram registradas a *strain* longitudinal global (SLG) e a *strain* circunferencial global (SCG). De acordo com o fluxograma do estudo ( Figura 1 ), os registros ecocardiográficos foram aceitos quando puderam ser realizados no período de uma semana a partir da avaliação por RMC. Todas as avaliações e cálculos ecocardiográficos foram realizados por um ecocardiografista experiente que desconhecia os achados clínicos, laboratoriais e de RMC do paciente.

### Avaliação por ressonância magnética cardíaca

Todas as avaliações de RMC foram realizadas em um scanner de 1,5 Tesla (Aera®; Siemens Healthineers, Erlangen, Alemanha). Os pacientes foram examinados com o disparo do eletrocardiograma usando uma bobina de corpo phased-array com 16 canais. Após a aquisição das imagens de varredura do localizador padrão, foram adquiridas as imagens cine em apneia nas visualizações de 2 e 4 câmaras dos ventrículos. Como agente de contraste, foi utilizada uma injeção intravenosa de 0,2 mmol/kg de Dotarem (gadoterato de meglumina; Guerbet LLC, Villepinte, França). Os exames de RMC foram avaliados por um radiologista que possui um certificado de imagem cardíaca com ampla experiência em RMC (> 9 anos). Os critérios atuais de Lake Louise foram usados para o diagnóstico de miocardite.^14^

O estudo foi realizado com a aprovação do comitê de ética local e o consentimento informado dos pacientes, de acordo com a Declaração de Helsinque.

### Análise estatística

As análises estatísticas foram realizadas com o software Statistical Package for Social Sciences 15.0 (SPSS, Chicago, IL, EUA). O teste de Kolmogorov–Smirnov foi realizado para avaliar se os dados tinham distribuição normal. As variáveis contínuas são apresentadas como média (desvio padrão) se a variável for distribuída como paramétrica, ou mediana (intervalo interquartil: Q1 a Q3) se a variável for distribuída como valores não paramétricos. As variáveis foram comparadas com os valores do teste t independente ou do teste de Mann–Whitney, dependendo do tipo de distribuição dos dados. As variáveis categóricas são apresentadas como números e porcentagens. Foram realizados o teste do qui-quadrado e o teste exato de Fisher para comparar as variáveis categóricas. O teste de correlação de Spearman foi usado para examinar a relação entre os valores de SLG, SCG e BNP. O valor preditivo, incluindo sensibilidade e especificidade de SLG e SCG para miocardite, foi determinado pela análise da curva do operador do receptor. Usando análise de regressão logística, foi determinada a associação entre SLG e SCG na miocardite. Além disso, SCG e SLG ajustados para idade na miocardite também foram avaliados por análise de regressão logística multivariada, uma vez que os pacientes com sequelas miocárdicas na RMC eram significativamente mais velhos. Valores de p < 0,05 foram considerados estatisticamente significativos.

## Resultados

Os pacientes foram separados em 2 grupos de acordo com os achados da RMC. Os pacientes do grupo RMC− (n = 21) não apresentaram sequelas miocárdicas na RMC, enquanto os do grupo RMC+ (n = 21) apresentaram sequelas miocárdicas.

Dados demográficos basais, comorbidades, contagem de hemoglobina, plaquetas, glóbulos brancos, neutrófilos, linfócitos, creatinina, TFG, níveis de proteína C-reativa, BNP, e troponina I, IMC, frequência cardíaca, PAS e PAD, como parâmetros associados aos valores da ES, são exibidos na Tabela 1 . Predomínio do sexo feminino, creatinina média, contagem mediana de glóbulos brancos, neutrófilos, linfócitos, plaquetas, hemoglobina, TFG, proteína C-reativa, PAD, IMC, e os valores médios de PAS e frequência cardíaca foram semelhantes e estatisticamente não significativos para ambos os grupos. A mediana de idade dos pacientes (maior nos pacientes com sequela miocárdica na RMC), a mediana da troponina I e os níveis de BNP foram diferentes e estatisticamente significativos no grupo de pacientes com sequela miocárdica na RMC ( Tabela 1 ).

**Tabela 1:** Parâmetros demográficos e laboratoriais de linha de base dos pacientes

	Pacientes com queixa de dor torácica		Valor p

	Sequelas miocárdicas na RMC–	Sequelas miocárdicas na RMC+	
Idade (anos), mediana (Q1-Q3)	43 (38-48)	46 (44-58)	0,03
Sexo feminino, n (%)	17 (81%)	13 (62%)	0,17
Creatinina (mg/dL), média ± DP	0,73±0,08	0,74±0,14	0,60
Glóbulos brancos (× 10^3^/L), mediana (Q1-Q3)	6,75 (6,56-8,74)	7,49 (6,51-8,54)	0,70
Neutrófilos (× 10^3^/L), mediana (Q1-Q3)	4,1 (3,24-5,43)	4,1 (3,34-5,66)	0,68
Linfócitos (× 10^3^/L), média ± DP	2,25±0,55	2,21±0,5	0,83
Plaquetas (× 10^3^/L), mediana (Q1-Q3)	261 (248-354)	265 (201-333)	0,23
Hemoglobina (g/dL), mediana (Q1-Q3)	13,4 (11-14)	12,7 (11,55-13,9)	0,94
TFG mediana (Q1-Q3)	102 (98-112)	100 (95-104)	0,35
Troponina I (ng/mL), mediana (Q1-Q3)	0,003 (0,001-0,003)	0,005 (0,002-1,35)	0,01
Proteína C-reativa, mediana (Q1-Q3)	1,95 (0,32-4,78)	1,09 (0,2-3,25)	0,31
BNP, mediana (Q1-Q3)	174 (127-222)	464 (404-470)	<0,001
Frequência cardíaca (bpm), média ± DP	70±4,4	70±4,4	0,73
PAS (mmHg), média ± DP	123±9	125±8	0,54
PAD (mmHg), mediana (Q1-Q3)	75 (62-75)	64 (61-69)	0,20
IMC (kg/m^2^), mediana (Q1-Q3)	23 (20-26)	23 (20-26)	0,67

*BNP: peptídeo natriurético cerebral; bpm: batimentos por minuto; dL: decilitro; DP: desvio padrão; g: grama; IMC: índice de massa corporal; kg: quilograma; L: litro; m: metro; min: minuto; mL: mililitro; mmHg: milímetro de mercúrio; PAD: pressão arterial diastólica; PAS: pressão arterial sistólica; RMC: ressonância magnética cardíaca; TFG: taxa de filtração glomerular.*

Os parâmetros ecocardiográficos como diâmetro da raiz aórtica, diâmetro do átrio esquerdo, diâmetro do septo interventricular, diâmetro da parede posterior, DDVE, DSVE, E, A, E’, A’, volume diastólico final e FEVE foram semelhantes em ambos os grupos, mas não estatisticamente significativos. Em contraste, os valores do volume sistólico final foram maiores, e os valores de S’, SCG e SLG foram menores nos pacientes com sequela miocárdica na RMC e significativamente semelhantes em ambos os grupos. Enquanto a FEVE, parâmetro ecocardiográfico tradicional mais utilizado, não apresentou significância estatística, os valores de SE, como SLG e SCG apresentaram. ( Tabela 2 ).

**Tabela 2:** Comparação dos parâmetros de ecocardiografia tradicional e de strain dos pacientes

	Pacientes com queixa de dor torácica		Valor p

	Sequelas miocárdicas na RMC–	Sequelas miocárdicas na RMC+	
RA (mm), mediana (Q1-Q3)	21 (20-21)	23 (19-24)	0,46
AE (mm), mediana (Q1-Q3)	33 (30-34)	32 (31-33)	0,79
SIV (mm), mediana (Q1-Q3)	10 (9-10)	10 (10-11)	0,18
PP (mm), mediana (Q1-Q3)	10 (9-10)	10 (10-11)	0,18
DDVE (mm), média ± SD	40±3,2	42±3,6	0,15
DSVE (mm), mediana (Q1-Q3)	27 (22-28)	28 (24-28)	0,18
E (cm/s), mediana (Q1-Q3)	86 (66-90)	74 (69-91)	0,85
A (cm/s), mediana (Q1-Q3)	62 (44-70)	69 (49-81)	0,21
E’ (cm/s), mediana (Q1-Q3)	9 (7-18)	10 (7-12)	0,81
A’ (cm/s), mediana (Q1-Q3)	9 (7,9-14)	10 (7,7-111)	0,33
S’ (cm/s), mediana (Q1-Q3)	9,5 (7,35-14)	7,8 (7,3-9)	0,03
VDF (mL), mediana (Q1-Q3)	63 (57,8-82)	73 (63-113)	0,06
VSF (mL), mediana (Q1-Q3)	19 (15-28)	23 (23-36)	0,01
FEVE (%), mediana (Q1-Q3)	70 (64-71)	68 (64-69)	0,05
SCG, mediana (Q1-Q3)	26,2 (27,8-25,1)	19 (21 -18,1)	<0,001
SLG, mediana (Q1-Q3)	25,6 (28,1-20,8)	20 (20,3-18,9)	<0,001

*A: velocidade da onda A; A’: velocidade da onda A’ lateral; AE: diâmetro do átrio esquerdo; cm: centímetro; DDVE: diâmetro diastólico do ventrículo esquerdo; DSVE: diâmetro sistólico do ventrículo esquerdo; E: velocidade da onda E; E’: velocidade da onda E’ lateral; FEVE: fração de ejeção do ventrículo esquerdo; mL: mililitro; mm: milímetro; PP: diâmetro da parede posterior; Q1-Q3: intervalo interquartil; RA: diâmetro da raiz aórtica; RMC: ressonância magnética cardíaca; s: segundo; S’: velocidade da onda S’ lateral; SCG: strain circunferencial global; SIV: diâmetro do septo interventricular; SLG: strain longitudinal global; VDF: volume diastólico final; VSF: volume sistólico final.*

**Figura 2:**
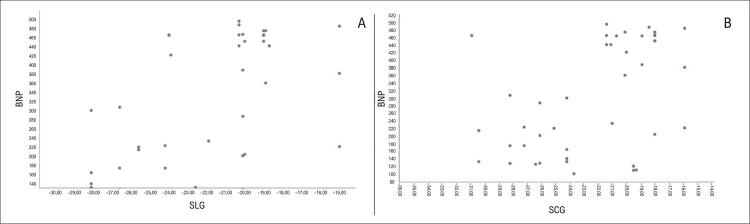
A correlação entre GLS e BNP (rho = 0,539, p < 0,001) (A), e a correlação entre GCS e BNP (rho = 0,429, p = 0,001) (B) são mostradas no diagrama de dispersão. BNP: peptídeo natriurético cerebral; SCG: strain circunferencial global; SLG: strain longitudinal global.

**Figura 3:**
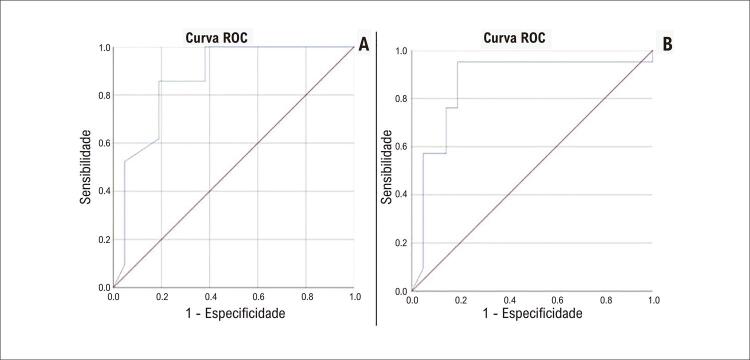
Conforme observado na análise da curva ROC, os valores de SLG apresentaram AUC de 0,866 com intervalo de confiança de 95% de 0,752 a 0,981 e p < 0,001 (A); e SCG apresentou um valor de AUC de 0,864 com intervalo de confiança de 95% de 0,736 a 0,992 e p < 0,001 (B). AUC: área sob a curva; ROC: característica de operação do receptor; SCG: strain circunferencial global; SLG: strain longitudinal global.

Houve correlação moderada entre SLG e BNP e, também, entre os valores de SCG e BNP ( Figura 2 ). Na análise multivariada ajustada por idade, os valores de SLG e SCG foram significativos, independentemente da idade ( Tabela 3 ). Conforme mostrado na Figura 3 para SLG e SCG, os valores da área sob a curva foram detectados como estatisticamente significativos.

**Tabela 3:** Associação entre SCG/SLG e miocardite (ajustada por idade) na análise multivariada

Variável	OR	IC de 95%		Valor p	Variável	OR	IC de 95%		Valor p
Idade	1,051	0,976	1,133	0,19	Idade	0,999	0,930	1,072	0,97
**SCG**	1,564	1,201	2,036	0,001	**SLG**	1,572	1,171	2,110	0,003

*IC: intervalo de confiança; OR: odds ratio; SCG: strain circunferencial global; SLG: strain longitudinal global.*

Um valor de SLG com ponto de corte < 20,35 mostrou sensibilidade de 85,7% e especificidade de 81%, e um valor de SCG com ponto de corte < 21,35 mostrou sensibilidade de 81% e especificidade de 81% na detecção de sequelas miocárdicas sem necessitar de avaliação por RMC ( Tabela 4 ).

**Tabela 4:** Pontos de corte preditivos de SLG e SCG para miocardite

Ponto de corte	Valor do SLG		Ponto de corte	Valor do SCG	
	
	Sensibilidade	Especificidade		Sensibilidade	Especificidade
**< 20,35**	85,7%	81%	**< 21,35**	81%	81%

*SCG: strain circunferencial global; SLG: strain longitudinal global.*

## Discussão

Até onde sabemos, este é o primeiro estudo a demonstrar que SLG e SCG são ferramentas valiosas para detectar sequelas de miocardite em pacientes com dor torácica como sintoma de COVID p/l após recuperação total da fase aguda de COVID-19.

Como problema de saúde pública, a COVID-19 é responsável por altas taxas de morbimortalidade em todo o mundo.^15^ As complicações cardiovasculares da COVID-19 também são responsáveis por essas taxas de morbimortalidade.^16^ A COVID-19 pode acometer o sistema cardiovascular a uma taxa de 20% com um espectro de piora do estado cardiovascular ou causando *de novo* complicações cardiovasculares. Diversas formas de complicações cardiovasculares podem ser categorizadas como lesão miocárdica, síndrome coronariana aguda ou exacerbação do estado cardiovascular.^17^ Essas patologias estão associadas a defeitos no suprimento/demanda de oxigênio, lesão mediada por citocinas, dano miocárdico direto mediado por vírus ou dano endotelial, instabilidade da placa e estado pró-trombótico de COVID-19.^18^

Em estudos populacionais de COVID-19, a dor torácica está presente em uma taxa menor do que na população geral, com incidência de 1,6% a 17,7%.^7 , 19^ Durante a fase aguda da COVID-19, a dor torácica pode ocorrer devido ao envolvimento cardíaco. Em alguns pacientes, a dor torácica pode continuar após a recuperação total da COVID-19, que é definida como a persistência dos sintomas da COVID-19 por um período > 3 a 4 semanas e é denominada “síndrome COVID p/l”.^20^

A dor torácica por lesão miocárdica pode ser detectada por altos níveis de troponina cardíaca,^21^ mas após a fase aguda da COVID-19, com COVID p/l, a RMC tem a capacidade de identificar de forma não invasiva o dano inflamatório do miocárdio e avaliar a gravidade do comprometimento funcional.^22^

O fato de a ecocardiografia ser uma ferramenta mais acessível e prática que a RMC significa que a ecocardiografia é mais viável nesses pacientes para os cardiologistas. Embora haja fortes evidências de envolvimento cardíaco de COVID-19 por RMC ou autópsia, a ecocardiografia tradicional pode detectar função sistólica normal na maioria dos pacientes.^23^

Além disso, alguns estudos mostraram que a ES pode ser usada para detectar disfunção ventricular em pacientes com COVID-19.^24 , 25^

A idade mediana da nossa população e a predominância de sexo foram semelhantes aos dados de Tudoran et al. ( Tabela 1 ).^26^ Entretanto, em nosso estudo, os pacientes com sequelas miocárdicas na RMC apresentaram mediana de idade mais elevada. Acreditamos que isso esteja relacionado ao fato de que o dano miocárdico se torna mais comum com a idade.

Em nosso estudo, os níveis de BNP e troponina I foram mais altos em pacientes com sequelas miocárdicas. Esses resultados estão de acordo com estudos recentes que indicam que concentrações mais altas de biomarcadores no sangue venoso, como creatina quinase isoenzima, mioglobina, troponina I e NT-proBNP, foram associados à gravidade da COVID-19 aguda mas não à COVID p/l.^27 - 29^Além disso, sabemos que o aumento do BNP é um marcador precoce de depressão miocárdica.^30^ O BNP é um indicador de dano miocárdico em modelos animais e está correlacionado com disfunção miocárdica.^31 , 32^ Ao contrário dos dados conhecidos de que níveis elevados de marcadores pró-inflamatórios, incluindo proteína C-reativa e linfopenia, têm sido associados com COVID p/l, os valores de proteína C-reativa e linfócitos foram estatisticamente semelhantes em nossos 2 grupos ( Tabela 1 ).^29^ Isso mostra que, nesses pacientes, as sequelas miocárdicas foram complicadas por disfunção miocárdica, e valores elevados de BNP foram associados a esses dados. Isso sugere que o dano miocárdico continua embora o processo inflamatório tenha terminado nos pacientes com envolvimento miocárdico por COVID p/l, e corrobora a correlação entre o nível de BNP e os valores de SCG-SLG em nosso estudo ( Figura 2 ). É importante diagnosticar esses pacientes rapidamente por ES e tratá-los para que o dano miocárdico não continue.

No presente estudo, IMC, frequência cardíaca, PAS e PAD foram semelhantes, o que pode afetar a avaliação por ES ( Tabela 1 ). Na RMC, o valor da FEVE ecocardiográfica tradicional foi estatisticamente não significativo e comparável entre pacientes com e sem sequela miocárdica. No entanto, os valores de SLG e SCG apresentaram forte diferença estatística e foram menores nos pacientes com sequela miocárdica na RMC ( Tabela 2 ).

Valores mais baixos de ES também foram relatados na fase aguda de COVID-19 por Bieber et al., Park et al., e Bhatia et al., e foi demonstrado que um valor de corte para SLG de 13,8, apesar da FEVE normal, estava associado com mortalidade significativamente maior durante a fase aguda da COVID-19.^12 - 13^ Essas informações mostram que os achados ecocardiográficos tradicionais não são comprometidos na COVID p/l. Em nosso estudo, também obtivemos valores de ES mais baixos em pacientes com COVID p/l. O valor do SLG com ponto de corte < 20,35 e o valor de SCG com ponto de corte < 21,35 apresentaram valor diagnóstico sem necessitar de avaliação por RMC para envolvimento miocárdico devido à COVID p/l ( Tabela 4 , Figura central ). Com base nesses valores, as sequelas miocárdicas podem ser detectadas de acordo com a RMC.

**Figura Central:**
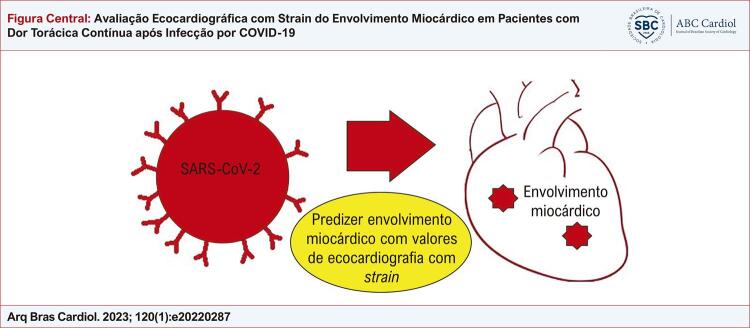
Avaliação Ecocardiográfica com Strain do Envolvimento Miocárdico em Pacientes com Dor Torácica Contínua após Infecção por COVID-19

A presença de lesão miocárdica pode ser detectada pela ES, que é tão valiosa quanto a RMC nesses pacientes. Considerando as desvantagens de custo-efetividade, acessibilidade e repetibilidade da RMC, bem como a facilidade de repetibilidade, custo-efetividade e fácil acessibilidade da ES no acompanhamento do processo de recuperação desses pacientes, a ES pode ser um método norteador para cardiologistas.

### Limitações

A limitação do nosso estudo é que foi retrospectivo e unicêntrico.

## Conclusão

A avaliação do envolvimento miocárdico na COVID p/l é mais complexa do que na fase aguda da COVID-19. Para evitar atrasos no tratamento na presença de envolvimento miocárdico, é importante diagnosticar os pacientes com sequelas miocárdicas com rapidez e precisão. Os cardiologistas, que são os principais profissionais no tratamento das doenças cardíacas, devem ter em mente que esses pacientes podem ser diagnosticados tanto com a ES quanto com a RMC. Nesse caso, os problemas de custo e repetibilidade da RMC podem tornar a ES uma ferramenta melhor para o diagnóstico e acompanhamento desses pacientes.
