# Supplementary material for: Avaliação Ecocardiográfica com Strain do Envolvimento Miocárdico em Pacientes com Dor Torácica Contínua após Infecção por COVID-19
Source: Arq Bras Cardiol. 2022 Dec 20;120(1):e20220287. [Article in Portuguese] doi: 10.36660/abc.20220287 (PMC9833213; doi:10.36660/abc.20220287)
Supplement: Translation

# Translation: Strain Echocardiographic Evaluation of Myocardial Involvement in Patients with Continuing Chest Pain after COVID-19 Infection

**Authors:** Emre Özdemir, Uğur Karagöz, Sadık Volkan Emren, Sedat Altay, Nihan Kahya Eren, Selin Özdemir, Mehmet Tokaç

**Affiliations:** 1 Turkey Department of Cardiology, Faculty of Medicine, Atatürk Research and Training Hospital, Katip Çelebi University, Izmir – Turkey; 2 Turkey Department of Cardiology, Torbalı Goverment Hospital, Izmir – Turkey; 3 Turkey Department of Radiology, Atatürk Research and Training Hospital, Izmir – Turkey; 4 Turkey Department of Infectious Diseases, Bozyaka Research and Training Hospital, Izmir – Turkey

**Keywords:** COVID-19, Echocardiography, Myocarditis

## Abstract

**Background:**

A new clinical manifestation called post or long coronavirus disease (p/l COVID) has walked into our lives after the acute COVID-19 phase. P/l COVID may lead to myocardial injury with subsequent cardiac problems. Diagnosing these patients quickly and simply has become more important due to the increasing number of patients with p/l COVID.

**Objectives:**

We compared strain echocardiography (SE) parameters of patients who suffered from atypical chest pain and had sequel myocarditis findings on cardiac magnetic resonance (CMR). We aimed to investigate the value of SE for detection of myocardial involvement in patients with p/l COVID.

**Methods:**

A total of 42 patients were enrolled. Our population was separated into two groups. The CMR(-) group (n = 21) had no myocardial sequelae on CMR, whereas the CMR(+) group had myocardial sequelae on CMR (n = 21). The predictive value of SE for myocarditis was also evaluated by age-adjusted multivariate analysis. P values < 0.05 were considered statistically significant.

**Results:**

When compared with left ventricular ejection fraction (LVEF), global longitudinal strain (GLS) and global circumferential strain (GCS) had a stronger relationship (LVEF, p = 0.05; GLS, p < 0.001; GCS, p < 0.001) with p/l COVID associated myocardial involvement. GLS < 20.35 had 85.7% sensitivity and 81% specificity; GCS < 21.35 had 81% sensitivity and 81% specificity as diagnostic values for myocardial sequelae detected with CMR. While there was no difference between the groups in terms of inflammatory markers (C-reactive protein, p = 0.31), a difference was observed between biochemical markers, which are indicators of cardiac involvement (brain natriuretic peptide, p < 0.001).

**Conclusion:**

SE is more useful than traditional echocardiography for making diagnosis quickly and accurately in order not to delay treatment in the presence of myocardial involvement.

## Introduction

In March 2020, the World Health Organization declared the novel coronavirus outbreak a global pandemic. We now know that COVID-19 causes not only viral pneumonia but also heart, vascular, cerebral, liver and kidney problems as a complex multisystem disease.^1 , 2^In the acute phase, cardiovascular involvement is caused by direct viral injury of the myocardium, multiple inflammatory injuries caused by cytokine storm, endothelial dysfunction due to vasculitis, destabilization of existing coronary plaques, pulmonary thromboembolism, microthrombogenesis, and injury caused by hypoxemia.^3 , 4^

However, some people still have symptoms, even after they have recovered from COVID-19, which is called p/l COVID syndrome.^5^In some series, chest pain has been reported in nearly 20% of patients after COVID-19 recovery.^6^The mechanism of chest pain is still unclear, but it could be linked to the long-term effects of COVID-19 on the myocardium.^7^Cardiac magnetic resonance (CMR) could play a role in the evaluation of this syndrome.^8^

Although strain echocardiography (SE) is not one of the routine echocardiographic procedures used by cardiologists, some studies have shown that low SE parameters can detect the progression of myocardial disease before traditional echocardiographic parameters become worse.^9 , 10^Low SE parameters can be detected during the acute phase of COVID-19 independently of clinical and traditional echocardiographic status and resolve during the follow-up period.^11 - 13^However, there are not enough data regarding the importance of SE parameters in the examination of patients with p/l COVID.

In this study, we evaluated the SE parameters of patients who suffered from atypical chest pain after they had fully recovered from COVID-19. We then compared these parameters with the CMR findings of myocarditis sequelae and investigated the value of SE for detecting myocardial involvement in patients with p/l COVID-19.

## Materials and methods

### Patient selection

In this study, we retrospectively scanned a total of 222 patients who underwent CMR evaluation due to any indication between February 2020 and December 2021 in a single center. In these patients, the period between the acute phase of COVID-19 and CMR evaluation, previous cardiac history and presence of cardiac tests (coronary computed tomography, myocardial perfusion scintigraphy, exercise stress test) to exclude coronary artery disease associated chest pain and continuing chest pain complaints were scanned from hospital records.

One hundred and eighty patients were excluded because: 1) the period between the acute phase of COVID-19 and CMR evaluation was less than 3 months, or the period was more than 3 months, but there was no PCR positive COVID-19 test (n = 102); 2) no continuing chest pain (n = 51); 3) echocardiography could not be performed within a period of one week from CMR evaluation (n = 11); 4) no cardiac test to exclude coronary artery disease-related chest pain (n = 8); 5) lack of other data in hospital records (n = 8), as exhibited in Figure 1 .

**Figure 1:** Flow chart of the study. CMR: cardiac magnetic resonance; PCR: polymerase chain reaction.

Patients’ symptoms of the acute COVID-19 infection period were questioned during admission with continuing chest pain. All of the patients had fever, cough, and mild dyspnea without requiring hospitalization, and none of them described chest pain during the acute phase of COVID-19.

A total of 42 patients who complained of chest pain that continued after recovery from COVID-19 and had CMR on hospital records were enrolled. All patients had no other comorbid diseases. All patients’ routine hemogram, biochemical tests, strain parameters, and traditional echocardiographic parameters were recorded. These patients were divided into two groups according to CMR findings compatible with myocarditis sequelae. Myocardial sequelae were detected as a subepicardial or a mid-wall late gadolinium enhancement (LGE) pattern that was predominantly located in the basal to mid-lateral segments of the left ventricle.

### Data collection

Data from hospital records, including serum hemoglobin (Hb), platelet, white blood cell (WBC), neutrophil (Neu), lymphocyte (Lym) counts, creatinine (Cr), glomerular filtration rate (GFR), C-reactive protein (CRP), brain natriuretic peptide (BNP), cardiac troponin I (TI) levels, systolic blood pressure (SBP), diastolic blood pressure (DBP), and body mass index (BMI) were collected.

All echocardiographic data was obtained using a standard EPIQ 7C echocardiography machine (Philips Medical Imaging, Eindhoven, Netherlands). The left ventricular diastolic diameter (LVDD), left ventricular systolic diameter (LVSD), left atrial diameter (LA), interventricular septal diameter (IVS) and posterior wall diameter (Pw), mitral inflow waves as the peak early wave (E) and late filling (A) wave, mitral annulus tissue doppler waves as systolic annular (s’), early diastolic annular (e’) and late (a’) diastolic annular velocities were assessed. Left ventricular ejection fraction (LVEF) was measured using the biplane Simpson’s method.

### Strain echocardiographic evaluation

Adequate echocardiographic data were accepted, with records saved at the end of exhalation, acquired from the peak of the R-wave, and all views from the apical 4-, 3-, and 2-chamber windows, as well as parasternal short axis from the basal, midventricular, and apical levels, assessed at a frame rate of 50 to 90 per second. The averages of 3 cardiac cycles were analyzed. The deformation parameters of all segments were calculated by the software (QLAB, Philips). Subsequently, global longitudinal strain (GLS) and circumferential strain (GCS) were noted. According to the study’s flow chart ( Figure 1 ), echocardiography records were accepted if they could be performed in a period of one week from the CMR evaluation. All echocardiographic evaulations and calculations were performed by an experienced echocardiographer who was not aware of the patient’s clinical, laboratory, and CMR findings.

### Cardiac magnetic resonance evaluation

All CMR evaluations were performed on a 1.5 Tesla scanner (Aera®; Siemens Healthineers, Erlangen, Germany). Patients were scanned with the electrocardiogram triggering using a 16-channel surface phased-array body coil. After standard localizer scan images were acquired, breath-hold cine images were acquired in the 2-chamber and 4-chamber views of the ventricles. As a contrast agent, an intravenous injection of 0.2 mmol/kg Dotarem (gadoterate meglumine; Guerbet LLC, Villepinte, France) was used. The CMR examinations were evaluated by a radiologist who has a cardiac imaging certificate with extensive CMR experience (> 9 years). Current Lake Louise criteria were used for the diagnosis of myocarditis.^14^

The study was performed with the approval of the local ethics committee and the informed consent of patients, according to the Declaration of Helsinki.

### Statistical analysis

Statistical analyses were performed with the Statistical Package for Social Sciences 15.0 software (SPSS, Chicago, IL, USA). The Kolmogorov–Smirnov test was performed to assess whether the data had a normal distribution. Continuous variables are presented as mean (standard deviation) if the variable is distributed as parametric, or median (interquartile range: Q1 to Q3) if the variable is distributed as non-parametric values. Variables were compared with independent t-test or Mann–Whitney test values depending on the type of data distribution. Categorical variables are presented as numbers and percentages. Chi-square test and Fisher’s exact test were performed to compare categorical variables. The Spearman’s correlation test was used to examine the relationship between GLS, GCS, and BNP values. The predictive value, including sensitivity and specificity of GLS and GCS for myocarditis, was determined by receiver operator curve analysis. Using logistic regression analysis, the association between GLS and GCS in myocarditis was determined. In addition, age-adjusted GCS and GLS in myocarditis were also evaluated by multivariate logistic regression analysis, since the patients with myocardial sequelae on CMR were statistically significantly older. P values < 0.05 were considered statistically significant.

## Results

The patients were separated into two groups according to CMR findings. The patients in the CMR− group (n = 21) had no myocardial sequelae on CMR, whereas those in the CMR+ group (n = 21) had myocardial sequelae.

Baseline demographics, comorbidities, Hb, Plt, WBC, Neu, Lym counts, Cr, GFR, CRP, BNP, TI levels, BMI, heart rate (HR), SBP, and DBP as parameters associated with SE values are shown in Table 1 . Female gender predominance, mean Cr, median WBC, Neu, Lym, Plt, Hb, GFR, CRP, DBP, BMI, and mean SBP and HR values were similar and statistically non-significant for both groups. The median age of the patients (higher in patients with myocardial sequelae on CMR), median TI, and BNP values were different and statistically significant in the group of patients with myocardial sequelae on CMR ( Table 1 ).

**Table 1:** Patients’ baseline demographic and laboratory parameters

	Patients with chest pain complaint		p value

	Myocardial sequelae on CMR–	Myocardial sequelae on CMR+	
Age (years), median (Q1-Q3)	43 (38-48)	46 (44-58)	0.03
Female gender, n (%)	17 (81%)	13 (62%)	0.17
Creatinine (mg/dL), mean ± SD	0.73±0.08	0.74±0.14	0.60
WBC (× 103/L), median (Q1-Q3)	6.75 (6.56-8.74)	7.49 (6.51-8.54)	0.70
Neu (× 103/L), median (Q1-Q3)	4.1 (3.24-5.43)	4.1 (3.34-5.66)	0.68
Lym (× 103/L), mean±SD	2.25±0.55	2.21±0.5	0.83
Plt (× 103/L), median (Q1-Q3)	261 (248-354)	265 (201-333)	0.23
Hb (g/dL), median (Q1-Q3)	13.4 (11-14)	12.7 (11.55-13.9)	0.94
GFR, median (Q1-Q3)	102 (98-112)	100 (95-104)	0.35
TI (ng/mL), median (Q1-Q3)	0.003 (0.001-0.003)	0.005 (0.002-1.35)	0.01
CRP, median (Q1-Q3)	1.95 (0.32-4.78)	1.09 (0.2-3.25)	0.31
BNP, median (Q1-Q3)	174 (127-222)	464 (404-470)	<0.001
HR (bpm), mean±SD	70±4.4	70±4.4	0.73
SBP (mmHg), mean±SD	123±9	125±8	0.54
DBP (mmHg), median (Q1-Q3)	75 (62-75)	64 (61-69)	0.20
BMI (kg/m2), median (Q1-Q3)	23 (20-26)	23 (20-26)	0.67

*BMI: body-mass index; BNP: brain natriuretic peptide; bpm: beats per minute; CMR: cardiac magnetic resonance; CRP: C-reactive protein; DBP: diastolic blood pressure; dL: deciliter; g: gram; GFR: glomerular filtration rate; Hb: hemoglobin; HR: heart rate; kg: kilogram; L: liter; Lym: lymphocytes; min: minute; mL: milliliter; mmHg: millimeter mercury; Neu: neutrophils; Plt: platelets; SBP: systolic blood pressure; SD: standard deviation; TI: troponin I; WBC: white-blood cells.*

Echocardiographic parameters such as aortic root diameter, LA, IVS, Pw, LVDD, LVSD, E, A, E’, A’, end-diastolic volume, and LVEF values were statistically non-significantly similar in both groups. In contrast, end-systolic values were higher, and S’, GCS, and GLS values were lower in the patients with myocardial sequelae on CMR and statistically significantly similar in both groups. While LVEF, the most commonly used traditional echocardiography parameter, showed no statistical significance, SE values such as GLS and GCS did ( Table 2 ).

**Table 2:** Comparison of patients’ traditional and strain echocardiography parameters

	Patients with chest pain complaint		p value

	Myocardial sequelae on CMR–	Myocardial sequelae on CMR+	
AR (mm), median (Q1-Q3)	21 (20-21)	23 (19-24)	0.46
LA (mm), median (Q1-Q3)	33 (30-34)	32 (31-33)	0.79
IVS (mm), median (Q1-Q3)	10 (9-10)	10 (10-11)	0.18
Pw (mm), median (Q1-Q3)	10 (9-10)	10 (10-11)	0.18
LVDD (mm), mean ± SD	40±3.2	42±3.6	0.15
LVSD (mm), median (Q1-Q3)	27 (22-28)	28 (24-28)	0.18
E (cm/s), median (Q1-Q3)	86 (66-90)	74 (69-91)	0.85
A (cm/s), median (Q1-Q3)	62 (44-70)	69 (49-81)	0.21
E’ (cm/s), median (Q1-Q3)	9 (7-18)	10 (7-12)	0.81
A’ (cm/s), median (Q1-Q3)	9 (7.9-14)	10 (7.7-111)	0.33
S’ (cm/s), median (Q1-Q3)	9.5 (7.35-14)	7.8 (7.3-9)	0.03
EDV (mL), median (Q1-Q3)	63 (57.8-82)	73 (63-113)	0.06
ESV (mL), median (Q1-Q3)	19 (15-28)	23 (23-36)	0.01
LVEF (%), median (Q1-Q3)	70 (64-71)	68 (64-69)	0.05
GCS, median (Q1-Q3)	26.2 (27.8-25.1)	19 (21 -18.1)	<0.001
GLS, median (Q1-Q3)	25.6 (28.1-20.8)	20 (20.3-18.9)	<0.001

*A: A-wave velocity; A’: lateral A’-wave velocity; AR: aortic root diameter; cm: centimeter; CMR: cardiac magnetic resonance; E: E-wave velocity; E’: lateral E’-wave velocity; ESV: end-systolic volume; GCS: global circumferential strain; GLS: global longitudinal strain; IVS: interventricular septum diameter; LA: left atrial diameter; LVDD: left ventricular diastolic diameter; LVEF: left ventricular ejection fraction; LVSD: left ventricular systolic diameter; mL: milliliter; mm: millimeter; Pw: posterior wall diameter; Q1-Q3: interquartile range; s: second; S’: lateral S’-wave velocity.*

There was a moderate correlation between GLS and BNP and also between GCS and BNP values ( Figure 2 ). In age-adjusted multivariate analysis, GLS and GCS values were found to be significant regardless of age ( Table 3 ). As shown in Figure 3 for GLS and GCS, the values for area under the curve were detected as statistically significant.

**Figure 2:** Correlation between GLS and BNP (rho = 0.539, p < 0.001) (A), and correlation between GCS and BNP (rho = 0.429, p = 0.001)(B) are shown on the scatter plot diagram. BNP: brain natriuretic peptide; GCS: global circumferential strain; GLS: global longitudinal strain.

**Table 3:** Association between GCS/GLS and myocarditis (adjusted by age) on multivariate analysis

Variable	OR	95% CI		p value	Variable	OR	95% CI		p value
Age	1.051	0.976	1.133	0.19	Age	0.999	0.930	1.072	0.97
**GCS**	1.564	1.201	2.036	0.001	**GLS**	1.572	1.171	2.110	0.003

*CI: confidence interval; GCS: global circumferential strain; GLS: global longitudinal strain; OR: odds ratio.*

**Figure 3:** As seen on ROC curve analysis, the GLS values had an AUC of 0.866 with a 95% confidence interval 0.752 to 0.981 and p < 0.001 (A); and GCS had a value of AUC of 0.864 with a 95% confidence interval 0.736 to 0.992 and p < 0.001 (B). AUC: area under the curve, GCS: global circumferential strain; GLS: global longitudinal strain; ROC: receiver operating characteristic.

A GLS value with a cut-off point of < 20.35 showed 85.7% sensitivity and 81% specificity, and a GCS value with a cut-off point of < 21.35 showed 81% sensitivity and 81% specificity in detecting myocardial sequelae without requiring CMR evaluation ( Table 4 ).

**Table 4:** Predictive cut-off values of GLS and GCS for myocarditis

Cut-off point	GLS value		Cut-off point	GCS value	
	
	Sensitivity	Specificity		Sensitivity	Specificity
**< 20.35**	85.7%	81%	**< 21.35**	81%	81%

*GCS: global circumferential strain; GLS: global longitudinal strain.*

## Discussion

To the best of our knowledge, this is the first study to demonstrate that GLS and GCS are valuable tools for detecting myocarditis sequelae in patients with chest pain as a symptom of p/l COVID after total recovery from the acute phase of COVID-19.

As a public health problem, COVID-19 is responsible for high rates of morbidity and mortality all over the world.^15^Cardiovascular complications of COVID-19 are also responsible for these morbidity and mortality rates.^16^COVID-19 can affect the cardiovascular system at a rate of 20% with a spectrum of worsening of cardiovascular status or causing de novo cardiovascular complications. Several forms of cardiovascular complications can be categorized as myocardial injury, acute coronary syndrome or exacerbation of cardiovascular status.^17^These pathologies are associated with oxygen supply/demand defects, cytokine-mediated injury, virus-mediated direct myocardial damage or endothelial damage, plaque instability, and prothrombotic status of COVID-19.^18^

In COVID-19 population studies, chest pain is present at a lower rate than in the general population, with an incidence of 1.6% to 17.7%.^7 , 19^During the acute phase of COVID-19, chest pain can occur due to cardiac involvement. In some patients, chest pain can continue after total recovery from COVID-19, which is defined as the persistence of COVID-19 symptoms for > 3 to 4 weeks and is named “p/l COVID syndrome.”^20^

Chest pain due to myocardial demage can be detected with high cardiac troponin levels,^21^but after the acute phase of COVID-19, with p/l COVID, CMR has the ability to identify non-invasively the inflammatory damage of the myocardium, assessing the severity of functional impairment.^22^

The fact that echocardiography is a more accessible and practical tool than CMR means that echocardiography is more feasible in these patients for cardiologists. Although there is strong evidence of cardiac involvement of COVID-19 by CMR or autopsy, normal systolic function can be detected by traditional echocardiography in most patients.^23^

Furthermore, some studies have shown that SE can be used to detect ventricular dysfunction in patients with COVID-19.^24 , 25^

Our population’s median age and gender predominance were similar to Tudoran et al. data ( Table 1 ).^26^However, in our study, the patients with myocardial sequelae on CMR had a higher median age. We believe that this is related to the fact that myocardial damage becomes more common with age.

In our study, BNP and TI levels were higher in patients with myocardial sequelae. These results are in accord with recent studies indicating that higher venous blood concentrations of biomarkers such as creatine kinase isoenzyme, myoglobin, troponin I and N-terminal probrain natriuretic peptide (NT-proBNP) were associated with the severity of acute COVID-19 but not p/l COVID.^27 - 29^Also, we know that BNP increases are an early marker of myocardial depression.^30^BNP is an indicator of myocardial damage in animal models and is correlated with myocardial dysfunction.^31 , 32^Contrary to the known data that elevated levels of pro-inflammatory markers, including CRP and lymphopenia, have been associated with p/l COVID, the CRP and Lym values were statistically similar in our two groups ( Table 1 ).^29^This shows that, in these patients, myocardial sequelae were complicated by myocardial dysfunction, and elevated BNP values were associated with these data. This suggests that myocardial damage continues even though the inflammatory process has ended in the patients with myocardial involvement of p/l COVID, and it supports the correlation between BNP level and GCS-GLS values in our study ( Figure 2 ). It is important to diagnose these patients quickly by SE and treat them so that myocardial damage does not continue.

In our study, BMI, HR, SBP, and DBP were similar, which can affect the SE evaluation ( Table 1 ). On CMR, the traditional echocardiographic LVEF value was statistically non-significant and comparable between patients with and without myocardial sequelae. However, GLS and GCS values had a strong statistical difference and were lower in patients with myocardial sequelae on CMR ( Table 2 ).

Lower SE values were also reported in the acute phase of COVID-19 by Bieber et al., Park et al., and Bhatia et al., and they demonstrated that a GLS cut-off value of 13.8, despite normal LVEF, was associated with significantly higher mortality during the acute phase of COVID-19.^12 - 13^This information shows that traditional echocardiographic findings are not impaired in p/l COVID. In our study, we also achieved lower SE values in p/l COVID. GLS value with a cut-off point of < 20.35 and GCS value with a cut-off point of < 21.35 had a diagnostic value without any need for CMR evaluation for p/l COVID myocardial involvement ( Table 4 , Central Illustration ). Based on these values, myocardial sequelae can be detected in accordance with CMR.

**Central Illustration:** Strain Echocardiographic Evaluation of Myocardial Involvement in Patients with Continuing Chest Pain after COVID-19 Infection

The presence of myocardial damage can be detected by SE, which is as valuable as CMR in these patients. Considering the cost-effectiveness, accessibility, and repeatability disadvantages of CMR, as well as the ease of repeatability, cost-effectivity, and easy accessibility of SE in the follow-up of the recovery process in these patients, SE can be a guiding method for cardiologists.

### Limitations

The limitation of our study is that it was retrospective and single-center.

## Conclusion

Evaluation of myocardial involvement in p/l COVID is more complex than the acute phase of COVID-19. In order to avoid delays in treatment in the presence of myocardial involvement, it is important to diagnose patients with myocardial sequelae quickly and accurately. Cardiologists, the main health professionals who treat cardiac diseases, should keep in mind that these patients can be diagnosed with SE as well as CMR. In this case, the cost and repeatability problems of CMR may make SE a better tool for diagnosis and follow-up of these patients.
